# Analysis on relationship between extreme pathways and correlated reaction sets

**DOI:** 10.1186/1471-2105-10-S1-S58

**Published:** 2009-01-30

**Authors:** Yanping Xi, Yi-Ping Phoebe Chen, Ming Cao, Weirong Wang, Fei Wang

**Affiliations:** 1 School of computer science and technology, Fudan University, Shanghai, PR China; 2 Faculty of Science and Technology, Deakin University, Melbourne, Australia; 3 Department of Biochemistry, School of Life Sciences, Fudan University, Shanghai, PR China

## Abstract

**Background:**

Constraint-based modeling of reconstructed genome-scale metabolic networks has been successfully applied on several microorganisms. In constraint-based modeling, in order to characterize all allowable phenotypes, network-based pathways, such as extreme pathways and elementary flux modes, are defined. However, as the scale of metabolic network rises, the number of extreme pathways and elementary flux modes increases exponentially. Uniform random sampling solves this problem to some extent to study the contents of the available phenotypes. After uniform random sampling, correlated reaction sets can be identified by the dependencies between reactions derived from sample phenotypes. In this paper, we study the relationship between extreme pathways and correlated reaction sets.

**Results:**

Correlated reaction sets are identified for *E. coli *core, red blood cell and *Saccharomyces cerevisiae *metabolic networks respectively. All extreme pathways are enumerated for the former two metabolic networks. As for *Saccharomyces cerevisiae *metabolic network, because of the large scale, we get a set of extreme pathways by sampling the whole extreme pathway space. In most cases, an extreme pathway covers a correlated reaction set in an 'all or none' manner, which means either all reactions in a correlated reaction set or none is used by some extreme pathway. In rare cases, besides the 'all or none' manner, a correlated reaction set may be fully covered by combination of a few extreme pathways with related function, which may bring redundancy and flexibility to improve the survivability of a cell. In a word, extreme pathways show strong complementary relationship on usage of reactions in the same correlated reaction set.

**Conclusion:**

Both extreme pathways and correlated reaction sets are derived from the topology information of metabolic networks. The strong relationship between correlated reaction sets and extreme pathways suggests a possible mechanism: as a controllable unit, an extreme pathway is regulated by its corresponding correlated reaction sets, and a correlated reaction set is further regulated by the organism's regulatory network.

## Background

In the past decades, genome-scale metabolic networks capable of simulating growth have been reconstructed for about twenty organisms [1]. A framework for *constraint*-*based reconstruction and analysis *(COBRA) has been developed to model and simulate the steady states of metabolic networks [2-4]. As reviewed in the literature [5], COBRA has been successfully applied to studying the possible phenotypes. Thus, it has attracted many attentions and gets rapid progress.

The COBRA framework represents a metabolic network as a stoichiometric matrix **S**. With the homeostatic-steady-state hypothesis and fluxes boundaries, all allowable steady-state flux distributions are limited in a space which can be represented as

(1) $$\begin{array}{cc} Sv=0, & \begin{array}{cc} v_{i}^{min}\leq v_{i}\leq v_{i}^{max}, & i=1,...,n \end{array} \end{array}$$

where **S**_*m *× *n *_is the stoichiometric matrix for a network consisting of *m *metabolites and *n *fluxes and **v**_*n *× 1 _is a vector of the flux levels through each reaction in the system [6].

Given the reversibility of reactions, an internal reversible reaction can be decoupled into two separate reactions for the forward and reverse directions separately. It means all fluxes should take a non-negative value and the solution space is now a convex polyhedral cone in high-dimensional space [6,7]. This convex cone can be spanned by a set of *extreme pathways *(ExPa), (**p**^*i*^, *i *= 1, ..., *k*) [8,9]. Every possible steady-state flux distribution in the solution space may therefore be represented as a non-negative combination of *extreme pathways *(ExPa):

(2) $$\begin{array}{cc} v=\sum_{i=1}^{k}\alpha_{i}p^{i}, & \begin{array}{cc} \alpha_{i}\geq 0, & \forall i \end{array} \end{array}$$

*Extreme pathways *(ExPa) have the following properties which make them biologically meaningful [10,11]:

1. The ExPa set of a given network is unique.

2. Each ExPa uses least reactions to be a functional unit.

3. The ExPa set is systemically independent which means an ExPa can't be decomposed into a non-negative combination of the remaining ExPas.

A similar network-based pathway definition as ExPa is *elementary flux modes *(EM) [12-14]. The algorithm for *elementary flux modes *(EM) treats internal reversible reactions differently from that for ExPas. Actually, ExPa set is a systemically independent subset of *elementary flux modes *(EM) and each EM can be represented by a non-negative combination of ExPas. The relationship and difference between ExPa and EM have been studied and articulated in literatures [10,15].

ExPas and EMs lead to a systems view of network properties [16] and they also provide a promising way to understand network functionality, robustness as well as regulation [17,18]. However, the number of ExPas for a reaction network grows exponentially with network size which makes the use of ExPas for large-scale networks computationally difficult [19,20].

A rapid and scalable method to quantitatively characterize all allowable phenotypes of genome-scale networks is uniform random sampling [21]. It studies the contents of the available phenotypes by sampling the points in the solution space. The set of flux distributions obtained from sampling can be analyzed to measure the pairwise correlation coefficients between all reaction fluxes and can be further used to define *correlated reaction sets *(CoSet). *Correlated reaction sets *(CoSet) are unbiased, condition-dependent definitions of modules in metabolic networks in which all the reactions have to be co-utilized in precise stoichiometric ratios [22]. It means the fluxes of the reactions in the same *correlated reaction sets *(CoSet) go up or down together in fixed ratios. We may think about whether CoSets provide clues about regulated procedures of a metabolic network.

Both ExPas and CoSets are determined by the topology of a metabolic network. Although lots of analyses were done on them separately [23-25], few attention has been paid to the relationship between them. Here, our aim is to reveal the relationship between ExPas and CoSets. We select *Escherichia coli *core metabolic network, human red blood cell metabolic network and *Saccharomyces cerevisiae *metabolic network as examples to start our research.

## Results and discussion

### Escherichia coli core metabolic network

We use the *E. coli *core model published on the web site of UCSD's systems biology research group. It is "a condensed version of the genome-scale E. coli reconstruction and contains central metabolism reactions" [26]. Details of this model can also be found in a published book [27]. The network contains 62 internal reactions, 14 exchange reactions and a biomass objective function.

The computation of the extreme pathways for E. coli core model results in 7784 ExPas, in which 7748 are type I or II ExPas and 36 are type III ExPas (Calculation and classification of ExPas are discussed in Methods section). The type I and II ExPas will be focused on herein and the reason for neglecting type III ExPas will be explained in Methods section. Twenty CoSets are identified on this network based on pairwise correlation coefficients between all reaction fluxes and listed in table 1.

**Table 1:** CoSets of E. coli core model.

CoSet ID	CoSet Size	Reactions
1	4	ACKr, ACt2r, EX_ac(e), PTAr

2	3	G6PDH2r, GND, PGL

3	3	EX_for(e), FORt, PFL

4	3	D_LACt2, EX_lac_D(e), LDH_D

5	3	CYTBD, EX_o2(e), O2t

6	3	ADHEr, ETOHt2r, EX_etoh(e)

7	2	TALA, TKT1

8	2	ICL, MALS

9	2	GAPD, PGK

10	2	FUM, SUCD4

11	2	FBA, TPI

12	2	EX_pyr(e), PYRt2r

13	2	EX_h2o(e), H2Ot

14	2	EX_glc(e), GLCpts

15	2	ENO, PGM

16	2	CO2t, EX_co2(e)

17	2	AKGt2r, EX_akg(e)

18	2	AKGDH, SUCOAS

19	2	ADK1, PPS

20	2	ACONT, CS

This table lists all CoSets of E. coli core model. We give each CoSet an ID and list it in the First column. We list CoSet size and reactions it contained in the second and third column. Reaction names are in abbreviated form. The abbreviation list is in table 7 and additional file 1.

**Figure 1:**
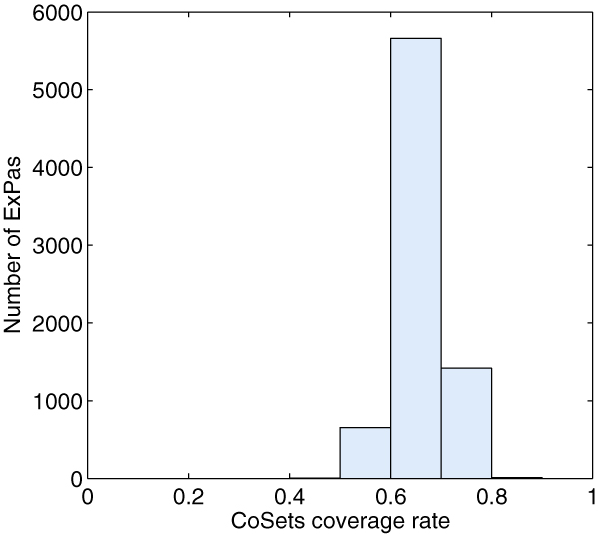
**CoSets coverage rate of ExPas of E. coli core metabolic network**. The y-axis indicates the number of extreme pathways which have the corresponding CoSets coverage rates; the x-axis lists the Cosets coverage rates, ranging from 0 to 1.

For each CoSet **C**_*j*_, we check how many type I and II ExPas use *k *reactions in **C**_*j*_, where *k *ranges from zero to the size of **C**_*j*_. The result is shown in table 2. Taking CoSet 3 as an example, from table 1 and 2, we can find that 3 reactions ('EX_for(e), FORt, PFL') belong to CoSet 3. Among all the type I and II ExPas, 5026 of them use all of these 3 reactions and 2722 use none of them. No ExPa uses one or two reactions. It is clear that each ExPa of *E. coli *core model covers in each CoSet in an 'all or none' manner. We also calculate, for each ExPa **p**^*i*^, the ratio of reactions in any CoSet which is fully covered by **p**^*i *^to all reactions in **p**^*i*^. The distribution of the ratios is shown in Figure 1. Each ExPa of *E. coli *core model covers at least one CoSet. The coverage rates are higher than 40% which implies that ExPas are under well control of CoSets.

**Table 2:** Relationship between ExPas and CoSets for E. coli core metabolic network.

CoSet ID	CoSet Size	Number of ExPas using k reactions of a CoSet				
		
		0	1	2	3	4
1	4	6652	0	0	0	1096

2	3	3556	0	0	4192	-

3	3	2722	0	0	5026	-

4	3	7151	0	0	597	-

5	3	1306	0	0	6442	-

6	3	3984	0	0	3764	-

7	2	3556	0	4192	-	-

8	2	5223	0	2525	-	-

9	2	928	0	6820	-	-

10	2	2240	0	5508	-	-

11	2	1352	0	6396	-	-

12	2	7106	0	642	-	-

13	2	1983	0	5765	-	-

14	2	904	0	6844	-	-

15	2	928	0	6820	-	-

16	2	1697	0	6051	-	-

17	2	6499	0	1249	-	-

18	2	5671	0	2077	-	-

19	2	5181	0	2567	-	-

20	2	2193	0	5555	-	-

This table illustrates relationship between ExPas and CoSets for *E. coli *core metabolic network. For each CoSet, we calculate how many ExPas cover *k *reactions in it where *k *ranges from 0 to size of this CoSet.

### Human red blood cell metabolic network

**Figure 2:**
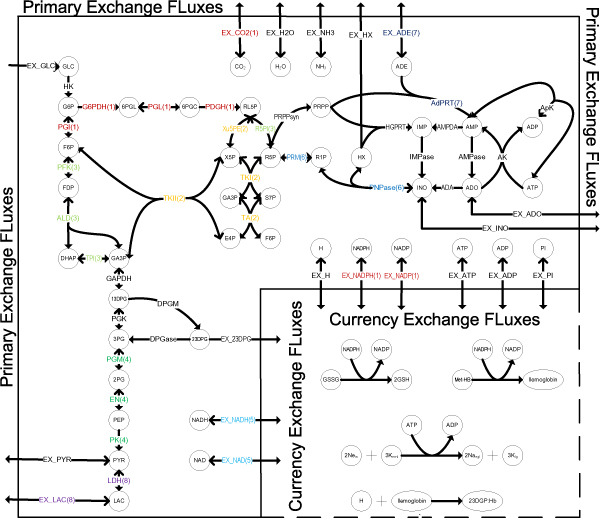
**Metabolic maps of RBC**. The graph is adapted from [25]. CoSet label of each reaction is added and different symbols are used to represent forward(→) and reverse(→) directions separately.

Human *red blood cell *(RBC) metabolic network has been well reconstructed and simulated [28-31]. The RBC model consists 39 metabolites, 32 internal metabolic reactions (See additional file 2) as well as 19 exchange fluxes (Figure 2) [25].

There are 55 ExPas calculated from the stoichiometric matrix of RBC model, among which 39 are type I or II ExPas and the others are type III ExPas. We focus on type I and II ExPas only. Type I and II ExPas are described in additional file 2. Eight CoSets are identified on RBC model. All CoSets are listed in table 3. The CoSets of RBC show agreement with the currently known regulatory structure [32]. There are 12 reactions regulated by inhibitors and activators or through post-translational modification. Most of them belong to some CoSet and most of CoSets have at least 1 regulated reaction. For example, regulated reactions 'G6PDH' and 'PDGH' belong to CoSet 1; 'TKI', 'TA' and 'TKII' belong to CoSet 2; 'RPI' and 'PFK' belong to CoSet 3; 'EN' and 'PK' belong to CoSet 4; 'AdPRT' belongs to CoSet 7. Although there's no regulated reaction in CoSet 6, it shares the metabolite 'R5P' with regulated reactions 'R5PI', 'TKI' and 'PRPPsyn'. So the reactions in CoSet 6 can be considered as being regulated indirectly. The other 2 reactions, 'PRPPsyn' and 'IMPase', don't belong to any CoSet.

**Table 3:** CoSets of RBC metabolic network.

CoSet ID	CoSets Size	Reactions
1	7	PDGH, Ex_CO2, Ex_NADPH, PGI, PGL, G6PDH, Ex_NADP

2	4	Xu5PE, TKI, TKII, TA

3	4	PFK, ALD, TPI, R5PI

4	3	PGM, EN, PK

5	2	Ex_NAD, Ex_NADH

6	2	PNPase, PRM

7	2	AdPRT, Ex_ADE

8	2	LDH, Ex_LAC

This table lists all CoSets of RBC model. We give each CoSet an ID and list it in the First column. We list CoSet size and reactions it contained in the second and third column. Reaction names are in abbreviated form. The abbreviation list is in table 7 and the list of internal reactions is in additional file 2.

The relationship between ExPas and CoSets is shown in table 4. Each CoSet is covered by an ExPa in an 'all or none' manner, except the CoSets 1 and 3. As for CoSets 1 and 3, some ExPas cover them in an 'all or none' manner and others cover them in 'one or all but one' mode. We look over the two exceptions to see which reactions are used by each ExPa and which are not used. As to CoSet 1, there are 24 ExPas covering it in an 'all or none' manner and 15 ExPas overlapping with it in a 'one or all but one' mode. Among these 15 ExPas, 6 ExPas use one and the same one reaction 'PGI' while other 9 ExPas use all the reactions in CoSet 1 only except the reaction 'PGI'. Similar situation can be found in CoSet 3. There are 12 ExPas overlapping with it in a 'one or all but one' mode, among which 6 ExPas use the same reaction 'R5PI' while other 6 ExPas cover all reactions but 'R5PI'.

**Table 4:** Relationship between ExPas and CoSets for RBC metabolic network.

CoSet ID	CoSets Size	Number of ExPas using k reactions of a CoSet							
		
		0	1	2	3	4	5	6	7
1	7	18	6	0	0	0	0	9	6

2	4	21	0	0	0	18	-	-	-

3	4	18	6	0	6	9	-	-	-

4	3	27	0	0	12	-	-	-	-

5	2	19	0	20	-	-	-	-	-

6	2	24	0	15	-	-	-	-	-

7	2	30	0	9	-	-	-	-	-

8	2	37	0	2	-	-	-	-	-

This table illustrates relationship between ExPas and CoSets for RBC metabolic network. For each CoSet, we calculate how many ExPas cover *k *reactions in it where *k *ranges from 0 to size of this CoSet.

The reasons for the complementary relationship on usage of reactions in CoSet 1 and CoSet 3 are as follows. 'PGI' belongs to one of 'historical' metabolic pathways named *Embden-Meyerhof-Parnas pathway *(EMP), while all other internal reactions in CoSet 1 are in pathway *Pentose Phosphate Pathway *(PPP). As for CoSet 3, 'R5PI' belongs to pathway PPP and all other reactions are in EMP. Since EMP provides the metabolite 'G6P' to PPP and inversely, PPP offers the metabolite 'GA3P' to EMP, the two pathways should cooperate with each other to fulfill the functions of the metabolic network. In order to work normally, the metabolic network may either utilize an ExPa using all the reactions in CoSet 1 (CoSet 3) or combine two (or more) ExPas together to fully cover CoSet1 (CoSet 3). By splitting some CoSet on different ExPas, it may bring redundancy and flexibility which are important properties for a cell to survive in various environments.

Both 'Ex_NADP' and 'Ex_NADPH' belong to CoSet 1, indicating the need of RBC cell to balance the NADPH/NADP ratio. According to "historically" partition of metabolic pathways, when pathway PPP is up-regulated, the quantity of NADP increases. When metabolic pathway EMP is up-regulated, the quantity of NADPH comes up. From the point of view of ExPa, 'Ex_NADP', 'Ex_NADPH' are used together in opposite direction by ExPas. It means that the fluxes through these reactions increase or decrease together. As a result, the quantity of NADP increases when that of NADPH decreases and vice versa. Situation is similar for reactions 'Ex_NAD' and 'Ex_NADH' in CoSet 5.

**Figure 3:**
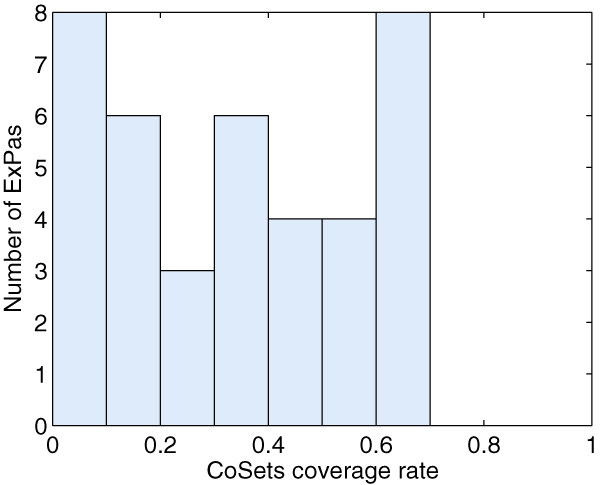
**CoSets coverage rate of ExPas of RBC metabolic network**. The y-axis indicates the number of ExPas which have the corresponding CoSets coverage rates; the x-axis represents the Cosets coverage rates, ranging from 0 to 1.

Figure 3 is the CoSets coverage rate of RBC model. Though the coverage rates are not as high as of those of *E. coli *core metabolic network, nearly 1/3 ExPas of RBC model has a CoSets coverage rate higher than 20%. There are 7 ExPas whose CoSets coverage rate is 0. All these 7 ExPas utilize relatively few reactions (1–3 internal reactions as well as the corresponding exchange reactions), among which, ExPas 10 and 11 utilize the regulated reaction 'IMPase', ExPas 12 and 13 are type II ExPas which serve to dissipate excess ATP, and ExPas 14, 15, 16 which participate in nucleotide metabolism may be regulated by the quantity of inosine and adenosine. In short, ExPas are in control of the regulatory structure of the metabolic network and our study suggests that the regulatory command usually spread from the regulated reactions to CoSets and finally to the related ExPas.

### Saccharomyces cerevisiae metabolic network

**Figure 4:**
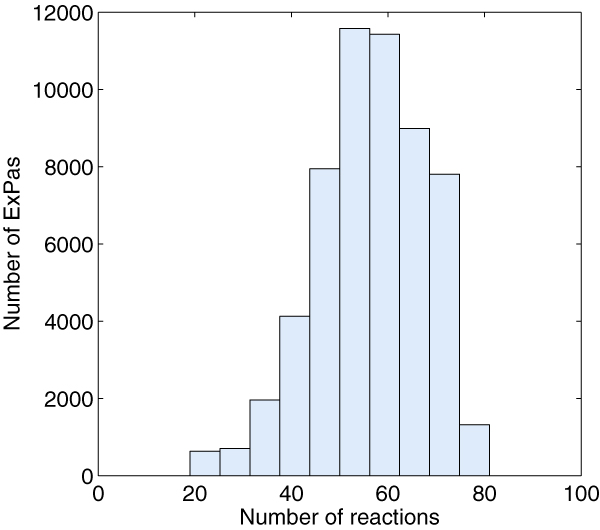
**Length of iND750's sample ExPas**. The y-axis indicates the number of ExPas which consist of the corresponding number of reactions; the x-axis represents the number of reactions contained in a single ExPa. The ExPa sampling process found no ExPa whose length is less than 20 or more than 80.

A full compartmentalized genome-scale metabolic model for *S. cerevisiae*, iND750, has been reconstructed and validated in 2004 [33]. We use this model to represent the metabolism of *S. cerevisiae*. Model iND750 accounts for 646 metabolites, 1149 internal reactions as well as 116 exchange fluxes excluding the objective reaction. Since the scale of iND750 is too large, enumerating all the ExPas of the model is computational intractable. Thus we samples a subset of ExPas to represent the whole ExPas (See Methods Section). The sampling procedure has executed 1000 times with 250–300 internal reactions being randomly removed out every time and finally resulted a sample set of 56496 unique ExPas. The lengths of sample ExPas range from 20 to 80 (Figure 4). It is difficult to sample the ExPas containing more than 80 reactions within acceptable cost of time.

One hundred and thirty five CoSets have been identified for this model. Some CoSets, especially the CoSets containing more than 5 reactions, have no sample ExPa passing through as if they are forgotten by the metabolic network. We name them *CoSets of solitary island*. We have tried different methods, such as removing all reactions which cannot be reached from a certain *CoSet of solitary island*, to sample some ExPas passing through the 'solitary island' but in vain because the sampling procedures take too much time. It seems that, the reactions in a *CoSet of solitary island *together with the reactions related to them form a complex network, and ExPas usually have to take a long way to go from some exchange reactions to a *CoSet of solitary island *and finally reach other exchange reactions. Because of the network's complexity, there are many bypaths along the road which causes a combinatorial explosion. So a *CoSet of solitary island *is not really solitary, and it is not too few but too many ExPas passing through these CoSets that prevent the ExPas computation algorithm, one step of which is enumerating all possible combinatorial paths, from catching them.

**Figure 5:**
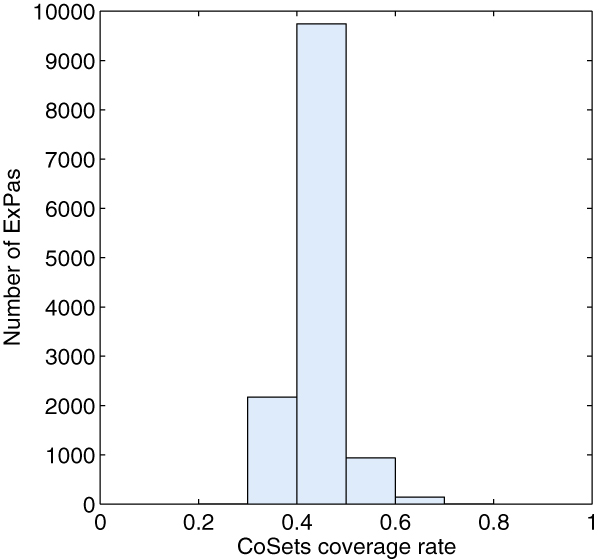
**CoSets coverage rate of ExPas of S. cerevisia model**. The y-axis indicates the number of extreme pathways which have the corresponding CoSets coverage rates; the x-axis lists the Cosets coverage rates, ranging from 0 to 1.

CoSets and the relationship between ExPas and CoSets are completely listed in additional files 4 and 5 separately. Due to the limited space, part of them are shown in table 5 and table 6. Figure 5 is the CoSets coverage rate distribution of S. cerevisiae model. We find that leaving out of the *CoSets of solitary island*, almost all the CoSets are covered by ExPas in an 'all or none' manner except CoSet 30 which is covered by ExPas in a complemental mode. CoSet 30 has three reaction members, 'AKGMAL', 'AKGt2r' and 'MALt2r'. Reaction 'AKGMAL' transports alpha ketoglutarate (AKG) and malate (MAL) across the epicyte in opposite directions. Reaction 'AKGt2r' transports AKG and hydrogen ion (H) across the epicyte in the same directions. And 'MALt2r' transports MAL and H across the epicyte in the same directions as well. If the quantity of AKG rises, the fluxes through 'AKGMAL' will grow up taking AKG and H out of the cell and bringing MAL into the cell. As a result, the quantity of H rises causing an increase on the flux of 'MALt2r'. Vice versa. These three reactions work together to balance the AKG/MAL ratio inside the cell and thus form a CoSet. Among the sample ExPas, we find that some of them utilize 'AKGMAL' and 'AKGt2r' while others use 'MALt2r' only. But, there are also some ExPas utilizing 'AKGt2r' while we don't find any sample ExPas that use the other two reactions in the CoSet. However, according to the above analysis, there should be some complemental ExPas utilizing reactions in the CoSet other than 'AKGt2r'. Otherwise, the cell will die due to the insupportable internal environment. Since the whole ExPa set is extremely large, the available ExPa sample set can only give a glance at the tremendous ExPa set and will certainly lose some information.

**Table 5:** CoSets of S. cerevisiae metabolic network.

CoSet ID	CoSet Size	Reactions
11	5	HETZK, HMPK1, PMPK, TMN, TMPPP

13	5	ACGKm, ACOTAim, AGPRim, ORNTACim, ORNt3m

20	3	PGCD, PSERT, PSP_L

22	3	GCCam, GCCbim, GCCcm

25	3	CYTK2, DCTPD, NDPK7

27	3	CYOOm, CYOR_u6m, O2tm

29	3	ARGSL, ARGSSr, OCBTi

30	3	AKGMAL, AKGt2r, MALt2r

31	3	AKGDam, AKGDbm, SUCOASm

33	3	ACSm, ADK1m, PPAm

34	3	ACLSm, DHAD1m, KARA1im

35	3	ABTA, GLUDC, SSALy

38	3	34HPPt2m, TYRTAm, TYRt2m

This table lists the no solitary island CoSets of *S. cerevisiae *metabolic network model with set size no less than 3. We give each CoSet an ID and list it in the First column. We list CoSet size and reactions it contained in the second and third column. Reaction names are in abbreviated form. The abbreviation list is in table 7 and additional file 3.

**Table 6:** Relationship between ExPas and CoSets for S. cerevisiae model.

CoSet ID	CoSet Size	Number of ExPas using k reactions of a CoSet					
		
		0	1	2	3	4	5
11	5	56445	0	0	0	0	51

13	5	49250	0	0	0	0	7246

20	3	39967	0	0	16529	-	-

22	3	54670	0	0	1826	-	-

25	3	56393	0	0	103	-	-

27	3	9983	0	0	46513	-	-

29	3	56454	0	0	42	-	-

30	3	47180	8900	416	0	-	-

31	3	56132	0	0	364	-	-

33	3	53692	0	0	2804	-	-

34	3	47600	0	0	8896	-	-

35	3	41082	0	0	15414	-	-

38	3	39550	0	0	16946	-	-

This table illustrates relationship between ExPas and CoSets for *S. cerevisiae *metabolic network. The CoSets listed here correspond to those in Table 5. For each CoSet, we calculate how many ExPas cover *k *reactions in it where *k *ranges from 0 to size of this CoSet.

The scale of *S. cerevisia *metabolic network is much larger. However, complementary relationship on usage of reactions in a CoSet is repeated as that in *E. coli *core metabolic network and RBC metabolic network.

## Conclusion

In this study, we investigated the relationship between CoSets and ExPas on the *in-silicon *representations of three metabolic networks. These models are different in species and scale. However, the experiment on each model leads to similar results that ExPas show strong complementary relationship on the usage of reactions in the same CoSet. It implies that this kind of relationship may exist in most of organisms. Since both CoSets and ExPas are generated from the topology information of metabolic networks, this phenomenon may reflect some inherent properties resulting from the topology constraints composed on the networks.

Moreover, our study not only reveals the interesting relationship between CoSets and ExPas but also provides a new sight of how the metabolic network works and how it is adjusted. The strong relationship between CoSets and ExPas provides clues about regulated procedure of a metabolic network, thus suggests a possible mechanism of how a metabolic network transferring its phenotype from one steady state to another. As functional units, ExPas are in control of the regulatory structure of the metabolic network, and the regulatory command usually spreads from regulated reactions to CoSets and finally to the related ExPas. As fluxes through each ExPa change according to the regulatory orders from its corresponding CoSets, the flux distribution of the whole metabolic network transfers towards a new steady state. By interrogating the relationship between CoSets and ExPas, we can tell which ExPas are possible to be up (down) regulated caused by an increasing (decreasing) flux in a given CoSet. This result may help predict the function of regulatory factors acting on metabolism. However, in order to answer the question which ExPas are really regulated, more information should be considered, such as regulatory structure of the metabolic networks as well as kinetic and thermodynamic constraints, which will be our future work.

## Methods

### ExPas computation and classification

ExPas are computed by an open source tool, 'expa', developed by Steven L. Bell and Bernhard O. Palsson [34]. The exchange fluxes can be separated into two groups: primary exchange fluxes and currency exchange fluxes. Primary exchange fluxes are external fluxes and currency exchange fluxes are fluxes external to metabolism but internal to the cell [27]. ExPas can be divided into three categories according to their use of exchange fluxes [35]. Type I ExPas utilize primary exchange fluxes as well as currency exchange fluxes. Type II ExPas involve currency exchange fluxes only. Type III ExPas are solely internal cycles without any exchange fluxes. Since type III ExPas are thermodynamically infeasible [36], we neglect type III ExPas and only focus on those of type I and II.

### CoSets computation

The CoSets of each metabolic model is generated by COBRA toolbox, an integrated toolbox of functions which are useful for analysis and simulation of organism's metabolic behavior [22]. For each model, uniform random sampling has been done first in the condition of optimum growth and results in 100,000 unique sample flux distributions that are available to the network. Then, 10,000 samples have been randomly selected and used to measure the pairwise correlation coefficients between reactions. We set the threshold of square pairwise correlation coefficient to 1 - 1*e*^-8^while identifying CoSets of each metabolic network assuring that reactions in the same CoSets have strong correlation with each other. The procedure of CoSets identification has been carried out 20 times for each model and the results are quite stable.

### Sampling for ExPa subset

We randomly delete a few reactions in *S. cerevisiae*'s iND750 model, and enumerate all the ExPas of the sub network. Then, the dimensions of deleted reactions are inserted back with zeros to these ExPas. As proved in Theorem 1, the ExPa set derived from sampling is a subset of the whole ExPa set of iND750. One thousand ExPa sets of different sub networks of iND750 model have been generated and merged together without redundancy. The union of all these ExPas constitute the sample set of ExPas used in the analysis on *Saccharomyces cerevisiae *metabolic network.

**Theorem 1**. *Suppose G is a metabolic network and *ℙ *is the ExPa set of G, then for any sub network G', its ExPa set *ℙ' *is a subset of *ℙ.

*Proof*. We assume that the available steady state flux distribution (**v**) of *G *lies in the convex cone C:

**Sv **= **0**, *v*_*i *_≥ 0, *i *= 1, ..., *n*

Without loss of generality, we assume *G' *is generated from *G *by deleting reactions *v*_*k*_, *v*_*k*+1_, ..., *v*_*n*_, then the steady state flux distribution of *G' *lies in the convex cone $c^{\prime }$:

$$\begin{array}{cc} Sv^{\prime }=0, & \{\begin{array}{ll} {v^{\prime }}_{i}\geq 0, & i=1,...,k-1\\ {v^{\prime }}_{i}=0, & i=k,...,n \end{array} \end{array}$$

Assuming that A = {**a**^*i *^| **a**^*i *^∈ C and $a_{j}^{i}$ = 0, *j *= *k*, ..., *n*}. Obviously, $A=c^{\prime }$.

∀**a**^*i *^∈ A, $\exists {\mathbb{P}}^{\prime \prime }\subseteq \mathbb{P}$, that

$$\begin{array}{ccc} a^{i}=\sum_{i=1}^{\left|{\mathbb{P}}^{\prime \prime }\right|}\alpha_{i}p^{i}, & \alpha_{i}\geq 0, & \forall i \end{array}$$

Since $a_{j}^{i}$ = 0, *j *= *k*, ..., *n*, then ∀**p**^*i *^∈ ℙ", $p_{j}^{i}$ = 0, *j *= *k*, ..., *n*, where **p**^*i *^is the *i*th ExPa in ℙ and $p_{j}^{i}$ is the *j*th component of **p**^*i*^.

Assuming that ℙ' = {**p**^*i *^| **p**^*i *^∈ ℙ and $p_{j}^{i}$ = 0, *j *= *k*, ..., *n*}. Thus, ${\mathbb{P}}^{\prime \prime }\subseteq {\mathbb{P}}^{\prime }$.

Because ${\mathbb{P}}^{\prime }\subseteq A$ and ℙ' is a systematically independent set, ${\mathbb{P}}^{\prime }\subseteq {\mathbb{P}}^{\prime \prime }$. Thus ℙ' = ℙ". Since the ExPa set of *G' *is unique, ℙ' is the ExPa set of *G'*, and ${\mathbb{P}}^{\prime }\subseteq \mathbb{P}$.   □

## List of abbreviations used

The abbreviations used in this study are listed in table 7.

**Table 7:** List of abbreviations used in this study.

**Concept Abbreviation**			
COBRA	Constraint-based reconstruction and analysis	EM	Elementary flux mode
CoSet	Correlated reaction set	RBC	Human Red Blood Cell
ExPa	Extreme pathway		

**Metabolite Abbreviation**			

AKG	Alpha ketoglutarate	MAL	Malate
GLC	Glucose	G6P	Glucose-6-phosphate
F6P	Fructose-6-phosphate	FDP	Fructose-1,6-phosphate
DHAP	Dihydroxyacetone phosphate	GA3P	Glyceraldehyde-3-phosphate
13DPG	1,3-Diphosphoglycerate	23DPG	2,3-Diphosphoglycerate
3PG	3-Phosphoglycerate	2PG	2-Phosphoglycerate
PEP	Phosphoenolpyruvate	PYR	Pyruvate
LAC	Lactate	6PGL	6-Phosphogluco-lactone
6PGC	6-Phosphogluconate	RL5P	Ribulose-5-phosphate
X5P	Xylulose-5-phosphate	R5P	Ribose-5-phosphate
S7P	Sedoheptulose-7-phosphate	E4P	Erythrose-4-phosphate
PRPP	5-Phosphoribosyl-1-pyrophosphate	IMP	Inosine monophosphate
R1P	Ribose-1-phosphate	HX	Hypoxanthine
INO	Inosine	ADE	Adenine
ADO	Adenosine	AMP	Adenosine monophosphate
ADP	Adenosine diphosphate	ATP	Adenosine triphosphate
NAD	Nicotinamide adenine dinucleotide	H	Hydrogen Ion
NADH	Nicotinamide adenine dinucleotide(R)	NH3	Ammonia
NADP	Nicotinamide adenine dinucleotide phosphate	Pi	Inorganic Phosphate
NADPH	Nicotinamide adenine dinucleotide phosphate(R)	CO2	Carbon Dioxide
H2O	Water		

**Pathway/Reaction Abbreviation**			

EMP	Embden-Meyerhof-Parnas pathway	PPP	Pentose Phosphate Pathway
34HPPt2m	3 4 hydroxyphenyl pyruvate mitochondrial transport via proton symport	ACKr	acetate kinase
ACOTAim	acteylornithine transaminase irreversible mitochondrial	ACONT	aconitase
ACt2r	acetate reversible transport via proton symport	ABTA	4 aminobutyrate transaminase
AGPRim	N acetyl g glutamyl phosphate reductase irreversible mitochondrial	ACSm	acetyl CoA synthetase
AKGDbm	oxoglutarate dehydrogenase dihydrolipoamide S succinyltransferase	ADHEr	Acetaldehyde dehydrogenase
ACGKm	acetylglutamate kinase mitochondrial	ALD	Aldolase
AKGDam	oxoglutarate dehydrogenase lipoamide	ADK1m	adenylate kinase mitochondrial
AdPRT	Adenine phosphoribosyl transferase	ADK1	adenylate kinase
AKGMAL	alpha ketoglutaratemalate transporter	AKGDH	2 Oxogluterate dehydrogenase
AKGt2r	2 oxoglutarate reversible transport via symport	ARGSL	argininosuccinate lyase
ARGSSr	argininosuccinate synthase reversible	ACLSm	acetolactate synthase mitochondrial
CYOR_u6m	ubiquinol 6 cytochrome c reductase	CS	citrate synthase
CYOOm	cytochrome c oxidase mitochondrial	CO2t	CO2 transporter via diffusion
CYTBD	cytochrome oxidase bd ubiquinol 8 2 protons	CYTK2	cytidylate kinase dCMP
D_LACt2	D lactate transport via proton symport	DCTPD	dCTP deaminase
DHAD1m	dihydroxy acid dehydratase 2 3 dihydroxy 3 methylbutanoate mitochondrial	EN	Enolase
ETOHt2r	ethanol reversible transport via proton symport	ENO	enolase
EX_ac(e)	Acetate exchange	EX_ADE	ADE exchange
EX_akg(e)	2 Oxoglutarate exchange	EX_co2(e)	CO2 exchange
EX_etoh(e)	Ethanol exchange	EX_for(e)	Formate exchange
EX_fum(e)	Fumarate exchange	EX_glc(e)	D Glucose exchange
EX_h2o(e)	H2O exchange	EX_LAC	LAC exchange
EX_lac_D(e)	D lactate exchange	EX_NAD	NAD exchange
EX_NADH	NADH exchange	EX_NADP	NADP exchange
EX_NADPH	NADPH exchange	EX_pyr(e)	Pyruvate exchange
FBA	fructose bisphosphate aldolase	EX_o2(e)	O2 exchange
FORt	formate transport via diffusion	G6PDH2r	glucose 6 phosphate dehydrogenase
GCCam	glycine cleavage complex lipoylprotein mitochondrial	GND	phosphogluconate dehydrogenase
GCCcm	glycine cleavage complex lipoylprotein mitochondrial	GLCpts	D glucose transport via PEPPyr PTS
GCCbim	glycine cleavage complex lipoylprotein irreversible mitochondrial	GLUDC	Glutamate Decarboxylase
GAPD	glyceraldehyde 3 phosphate dehydrogenase	HETZK	hydroxyethylthiazole kinase
GAPDH	Glyceraldehyde phosphate dehydrogenase	H2Ot	H2O transport via diffusion
HMPK1	hydroxymethylpyrimidine kinase ATP	ICL	Isocitrate lyase
KARA1im	acetohydroxy acid isomeroreductase mitochondrial	LDH	Lactate dehydrogenase
NDPK7	nucleoside diphosphate kinase ATPdCDP	MALS	malate synthase
MALt2r	L malate reversible transport via proton symport	LDH_D	D lactate dehydrogenase
O2t	o2 transport diffusion	O2tm	O2 transport diffusion
OCBTi	ornithine carbamoyltransferase irreversible	PFK	Phosphofructokinase
ORNTACim	ornithine transacetylase irreversible mitochondrial	PGM	Phosphoglyceromutase
ORNt3m	ornithine mitochondrial transport via proton antiport	PFL	pyruvate formate lyase
PGCD	phosphoglycerate dehydrogenase	PGI	Phosphoglucoisomerase
PGK	phosphoglycerate kinase	PGL	6 phosphogluconolactonase
PGL	6-phosphoglyconolactonase	PGM	phosphoglycerate mutase
PDGH	6-phosphoglycononate dehydrogenase	PMPK	phosphomethylpyrimidine kinase
PGPPAm_SC	phosphatidylglycerol phosphate phosphatase A yeast specific mitochondrial	PK	Pyruvate kinase
PNPase	Purine nucleoside phosphorylase	PPS	phosphoenolpyruvate synthase
PRM	Phosphoribomutase	PSERT	phosphoserine transaminase
PSP_L	phosphoserine phosphatase L serine	PTAr	phosphotransacetylase
PYRt2r	pyruvate reversible transport via proton symport	R5PI	Ribose-5-phosphate isomerase
SSALy	succinate semialdehyde dehydrogenase NADP	SUCD4	succinate dehyrdogenase
SUCOAS	succinyl CoA synthetase ADP forming	TA	Transaldolase
SUCOASm	Succinate CoA ligase ADP forming	TALA	transaldolase
TYRt2m	tyrosine mitochondrial transport via proton symport	TKII	Transketolase
TYRTAm	tyrosine transaminase mitochondrial	TKT1	transketolase
TMPPP	thiamine phosphate diphosphorylase	TMN	thiaminase
TPI	Triose phosphate isomerase	TKI	Transketolase
Xu5PE	Xylulose-5-phosphate epimerase		

Abbreviations used in this study are divided into three parts. They are concept abbreviations, metabolite abbreviations and pathway/reaction abbreviations.

## Competing interests

The authors declare that they have no competing interests.

## Authors' contributions

YX designed the system, implemented programs, carried out the analysis, and participated in manuscript preparation. YPC supervised the project and suggested ways of improving the study, and participated in writing the manuscript. MC helped implement programs. WW participated in discussion of the research. FW designed and directed the research, and drafted the manuscript. All authors read and approved the final manuscript.

## Supplementary Material

Additional file 2 **Maps of Reactions and ExPas of RBC metabolic network**. This is a PDF file with a table and a figure. The table describes all the internal reactions in RBC metabolic network and the figure shows all the type I and II ExPas of this model. Click here for file

Additional file 4 **All the CoSets of S. cerevisiae metabolic network**. This is an *Excel*^® ^file of all the 135 CoSets of *S. cerevisiae *metabolic network. Click here for file

Additional file 5 **Relationship between ExPas and CoSets for S. cerevisiae model (full version)**. This is an *Excel*^® ^file of relationship between ExPas and all the 135. CoSets for *S. cerevisiae *model. Click here for file

Additional file 1 **The reaction abbreviation list of E. coli core metabolic network**. This is an *Excel*^® ^file of reaction abbreviations and reaction names of *E. coli *core metabolic network. Click here for file

Additional file 3 **The reaction abbreviation list of S. cerevisiae metabolic network**. This is an *Excel*^® ^file of reaction abbreviations and reaction names of *S. cerevisiae *metabolic network. Click here for file
