# Supplementary material for: Analysis on relationship between extreme pathways and correlated reaction sets
Source: BMC Bioinformatics. 2009 Jan 30;10(Suppl 1):S58. doi: 10.1186/1471-2105-10-S1-S58 (PMC2648798; doi:10.1186/1471-2105-10-S1-S58)
Supplement: Additional file 2 — Maps of Reactions and ExPas of RBC metabolic network. This is a PDF file with a table and a figure. The table describes all the internal reactions in RBC metabolic network and the figure shows all the type I and II ExPas of this model. [file 1471-2105-10-S1-S58-S2.pdf]

Abbreviation	Chemical Reaction
**Glycolysis and Rapoport-Leubering Shunt**	
HK	$GLU + ATP \xrightarrow{\text{Hexokinas}} G6P + ADP + H$
PGI	$G6P \xleftrightarrow{\text{Phosphoglucoisomerase}} F6P$
PFK	$F6P + ATP \xrightarrow{\text{Phospofructokinas}} FDP + ADP + H$
ALD	$FDP \xleftrightarrow{\text{Aldolase}} GA3P + DHAP$
TPI	$GA3P + NAD + Pi \xleftrightarrow{\text{Triose phosphate isomerase}} 13DPG + NADH + H$
GAPDH	$GA3P + NAD + Pi \xleftrightarrow{\text{Glyceralderhyde phosphate dehydrogenase}} 13DPG + NADH + H$
PGK	$13DPG + ADP \xleftrightarrow{\text{Phosphoglycerate kinase}} 3PG + ATP$
DPGM	$13DPG \xrightarrow{\text{Diphosphoglyceromutase}} 23DPG + H$
DPGase	$23DPG + H2O \xrightarrow{\text{Diphosphoglycerate phosphatase}} 3PG + Pi$
PGM	$3PG \xleftrightarrow{\text{Phosphoglyceromutase}} 2PG$
EN	$2PG \xleftrightarrow{\text{Enolase}} PEP + H2O$
PK	$PEP + ADP + H \xrightarrow{\text{Pyruvate kinase}} PYR + ATP$
LDH	$PYR + NADH + H \xleftrightarrow{\text{Lactate dehydrogenase}} LAC + NAD$
**Pentose Phosphate Pathway**	
G6PDH	$G6P + NADP \xrightarrow{\text{Glucose−6−phosphate dehydrogenase}} 6PGL + NADPH + H$
PGL	$6PGL + H2O \xleftrightarrow{\text{6−phosphoglyconolactonase}} 6PGC + H$
PDGH	$6PGC + NADP \xrightarrow{\text{6−phosphoglyconate dehydrogenase}} RL5P + NADPH + CO2$
R5PI	$RL5P \xleftrightarrow{\text{Ribose−5−phosphate isomerase}} R5P$
Xu5PE	$RL5P \xleftrightarrow{\text{Xylulose−5−phosphate epimerase}} X5P$
TKI	$X5P + R5P \xleftrightarrow{\text{Transketolase}} S7P + GA3P$
TA	$GA3P + S7P \xleftrightarrow{\text{Transaldolase}} E4P + F6P$
TKII	$X5P + E4P \xleftrightarrow{\text{Transketolase}} F6P + GA3P$
**Adenosine Nucleotide Metabolism**	
PRPPsyn	$R5P + ATP \xrightarrow{\text{Phosphoribosyl pyrophosphate synthetase}} PRPP + AMP$
PRM	$R1P \xleftrightarrow{\text{Phosphoribomutase}} R5P$
HGPRT	$PRPP + HX + H2O \xrightarrow{\text{Hypoxanthine guanine phosphoryltransferase}} IMP + 2Pi$
AdPRT	$PRPP + ADE + H2O \xrightarrow{\text{Adenine phosphoribosyl transferase}} AMP + 2Pi$
PNPase	$INO + Pi \xleftrightarrow{\text{Purine nucleoside phosphorylase}} HX + R1P$
IMPase	$IMP + H2O \xrightarrow{\text{Inosine monophosphatase}} INO + Pi + H$
AMPDA	$AMP + H2O \xrightarrow{\text{Adenosine monophosphate deaminase}} IMP + NH3$
AMPase	$AMP + H2O \xrightarrow{\text{Adenosine monophosphate phosphohydrolase}} ADP + Pi + H$
ADA	$ADO + H2O \xrightarrow{\text{Adenosine deaminase}} INO + NH3$
AK	$ADO + ATP \xrightarrow{\text{Adenosine kinase}} ADP + AMP$
ApK	$2\ ADP \xleftrightarrow{\text{Adenylate kinase}} ATP + AMP$

Table 3 - The internal reaction in RBC model.
Reaction names are in abbreviated form. The abbreviation list is in appendix.

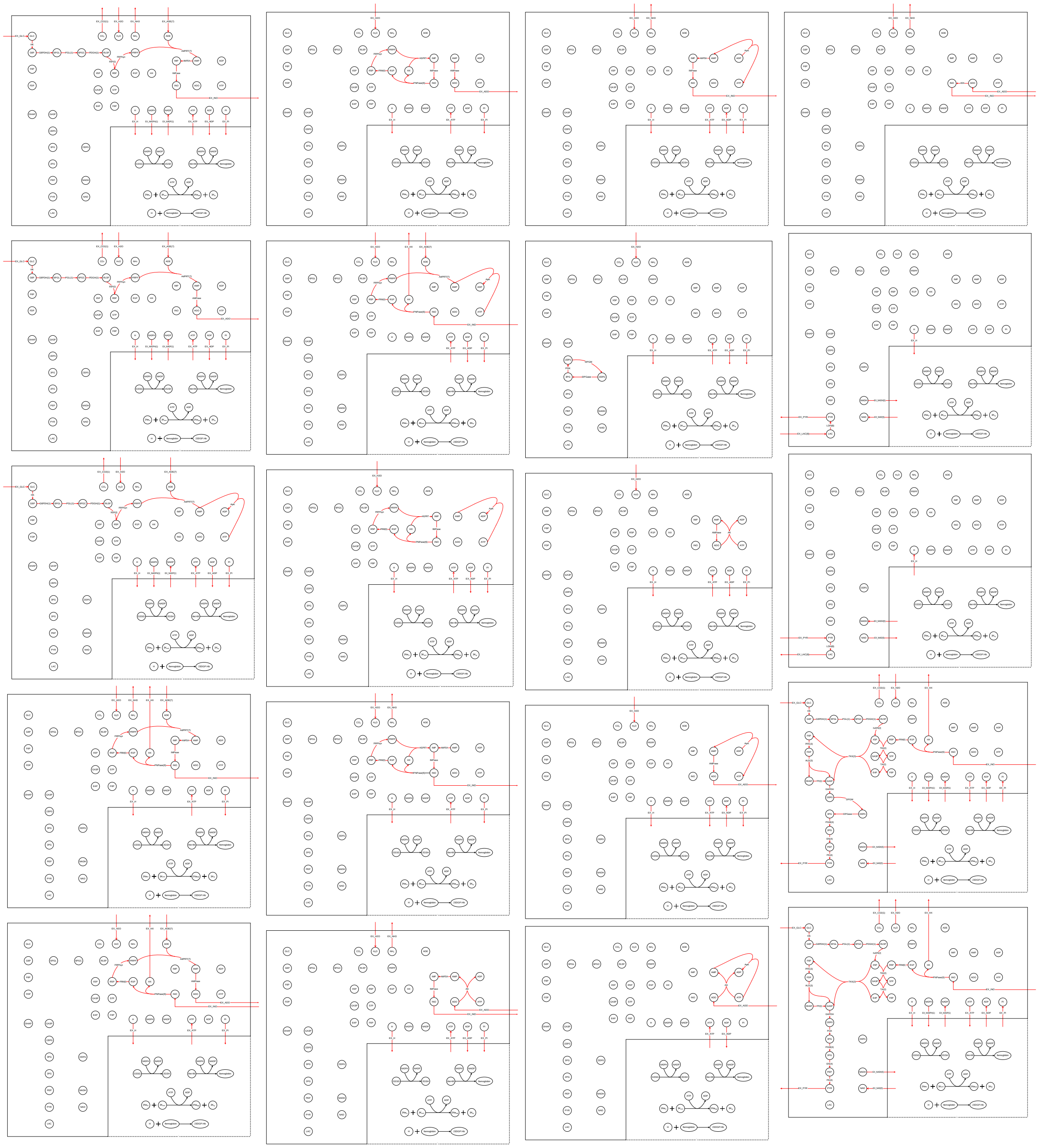

Figure 1: Steady-state flux maps for all type I and type II red blood cell extreme pathways. Extreme pathways are listed in column major order: the 1st to the 5th extreme pathways are in the first column, from top to bottom, the 6th to the 10th are in the second column, and so on.

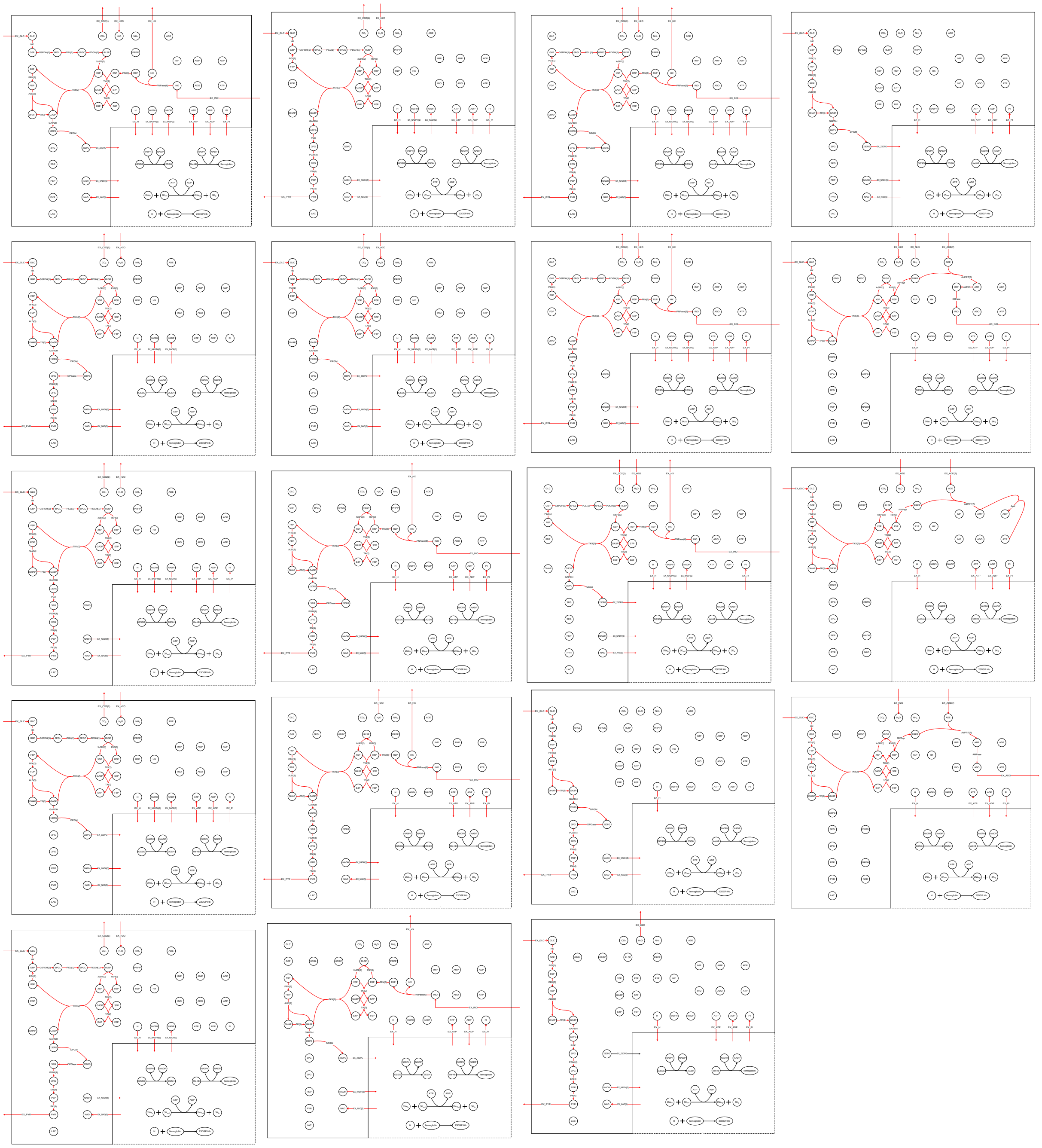

Figure 1: Continued. Steady-state flux maps for all type I and type II red blood cell extreme pathways. Extreme pathways are listed in column major order: the 21st to the 25th extreme pathways are in the first column, from top to bottom, the 26th to the 30th are in the second column, and so on.
